# Hospital and Institutional News

**Published:** 1914-08-22

**Authors:** 


### HOSPITAL AND INSTITUTIONAL NEWS.

### "THE HOSPITAL'S" SPECIAL CORRESPONDENTS.

The response to our request last week for someone to act as special correspondent to us at every hospital, especially on the East Coast, during the war, and with every Voluntary Aid Detachment, is bringing us letters, offers, and inquiries in large numbers. To supplement, therefore, our previous request for news of all the hospital arrangements in each institution and its locality which the war has occasioned, let us state briefly what the duties involve. The duties resolve themselves into one person making it his business *regularly* to send us—if possible, arriving here by the first post on *Monday* morning—an account of the progress made with the arrangements (the names of the staff) during the week; perhaps later to send us an account of the arrival and reception of the wounded. The great point is the regular posting to us by Monday morning of an account of the local hospital doings and changes during the preceding seven days. All such special correspondents will be paid like the other contributors to The Hospital, and the co-operation which made our last week's issue cover such a wide field shows what can be done with a little organising. What the hospitals are doing we want each Voluntary Aid Detachment to do also.

### A LONDON HOSPITAL SECRETARY LEAVES FOR ACTIVE SERVICE.

Mr. A. E. Thomas, the secretary of the Hampstead General and North-West London Hospital, has been called up and left on Sunday, August 9, to join his regiment, the Honourable Artillery Company. We believe that he has the distinction of being the first hospital secretary to leave for the front, and he takes with him the good wishes of all his colleagues. Mr. Thomas was on his holiday when the war broke out, having left on July 25. He had been away about ten days when he was hastily summoned back and only spent one night at the hospital *en route* to report himself to headquarters. Mr. Conrad W. Thies, the energetic co-secretary of the British Hospitals Association, and formerly secretary of the Royal Free Hospital, has kindly stepped into the breach, and volunteered, not for the front, but for the equally useful and necessary hospital work at home. A Hampstead resident himself, he has kindly taken over Mr. Thomas's work, and is now acting as honorary secretary of the Hampstead General Hospital.

### HOW EX-HOSPITAL MEN MAY HELP.

While Mr. A. E. Thomas appears to be the only hospital secretary, at any rate from London, who has left work for active service, the secretarial offices have been somewhat disorganised by the departure of clerks to join the Territorials. The Metropolitan Convalescent Institution has lost most of its clerical staff, other institutions have been equally affected; and this raises a practical question of much importance. The above gaps have to be filled somehow; skilled assistance is necessary. Surely the best means of providing this is for ex-hospital men, now on the retired list and too old for active service, to come forward, as Mr. Thies has done, and undertake honorary work. There are, we are glad to think, many ex-hospital officials of similar standing and experience who could, if they would, render invaluable assistance in this way. It is only two or three years ago since Mr. Nixon retired from University College Hospital, and recent news of him is, we are happy to think, that he is in excellent health. Mr. R. Owthwaite, who is now assisting Mr. Alexander Hayes with Mr. Thies's work as joint secretary for the British Hospitals Association, is an example of another hospital officer who has acted on this suggestion. He has also offered to help in any way that he can at his old institution, the City of London Hospital for Diseases of the Chest. No way could be more practical than this of experienced men coming forward to fill the gaps left by acting officials till the latter's return from active service, and we hope that all institutional workers on the retired list anxious to be of service will place themselves at once into communication with their old hospitals, if they have not done so already.

### THE POSITION AT THE GERMAN HOSPITAL.

The German Hospital has lost half its staff of doctors and several of its nurses. The matron of the hospital has stated that five of the medical staff had been taken for the war and that nine of the nurses who were on holiday in Germany had not returned. The hospital, however, is still open and has over 100 patients, including, as usual, several English, in the wards.

### POOR-LAW INFIRMARIES AND FOOD SUPPLIES: ASYLUMS BOARD'S DECISION.

The vast quantities of food stores and drugs required for the sick in Poor-Law institutions have exercised the minds of those responsible for their care during the past few days. The Metropolitan Asylums Board have held a special meeting to deal with this important question, and they ratified the action of the clerk in his communications to their contractors for foods and drugs. Whilst the moratorium does not apply to these contracts, it is generally felt that contractors who are endeavouring to fulfil their obligations should not be penalised through no fault of their own, and consequently it has been decided favourably to consider any extra cost that may be incurred by a contractor in procuring a constant supply of goods for the infirmaries and other institutions where the sick and helpless are cared for. It is most likely that additional patients will be placed under the care of the Guardians, as the hospitals in some instances have placed a number of their beds at the service of the War Office for the wounded. For instance, in consequence of the fact that St. Thomas's Hospital have set aside 200 beds they have notified the Lambeth Board of Guardians. The latter, therefore, will have to be prepared to take patients who in the ordinary course of events would have been treated in St. Thomas's Hospital. The Guardians of London, with few exceptions, have followed the example of the Metropolitan Asylums Board, and decided to treat their contractors in a generous manner.

### A BOARD ROOM AS A CHILDREN'S WARD.

It is not only war which has its emergencies, as the following facts will show at a hospital now in the transition stage from a small to a large institution. First, it is satisfactory to hear that from the current accounts a sum of £700 has been put aside as the nucleus of an extension fund in connection with the Pontypool and District Hospital. The needs of the moment—for the hospital's work has grown considerably of recent years—are a nurses' home, a children's ward, and an *x*-ray installation, the total cost of which is expected to amount to £4,000, with a balance of income on the year of over £400, for which congratulations are due to Mr. T. Morgan, the secretary; the financial position is hopeful, and Mr. George Jenkins, who presided at the half-yearly meeting, expects that the improvements will not have long to wait. As to the children's ward, for instance, they are housed in what was originally intended for a board room, and though, of course, this enables them to receive treatment which could not be carried out in their own homes, it is not satisfactory in the hospital sense. A further change in the purpose of this room will take place when the new children's ward is completed, for the ex-board room and former ward will then be used to accommodate the *x*-ray apparatus that is wanted. After the alterations have been made, there will be approximately fifty beds in the hospital.

### SUGGESTED DIVERSION OF SURPLUS PANEL FUNDS.

As indicative of the sentiments now agitating the public mind, we record the fact that Dr. Henderson, a medical practitioner of Southport, has suggested that the profession should surrender all the unallocated medical funds under the Insurance Act as a donation to the War Relief Fund. Without discussing the intention animating this proposal, it is necessary to record the fact that if Dr. Henderson's colleagues ever agreed to his suggestion, it would still be necessary for the Insurance Commissioners to authorise the dispersion of the unallocated funds, and in view of the fact that for whomever they were originally intended they were certainly not designed for a War Relief Fund, special powers would have to be acquired from Parliament for this purpose. Those professional men who agree, and those who disagree with this suggestion will probably, however, feel that such funds as the War Relief Fund should, as a matter of principle, be formed entirely by private generosity, and in a time when that is flowing freely it would be an extremely bad principle to divert any public funds to such purposes. We must never sacrifice patriotic impulses to common sense, but we quote this as a sample of the difficulties which such impulses when given full rein, create—and of the principles which they sacrifice in the process.

### THE PIVOT OF THE VOLUNTARY HOSPITAL.

The many offers of help make it plain that all the new and various requisites to make war for our defenders as secure as possible by the organisation of supplementary hospital units—*i.e.*, of establishments for the reception of the sick and wounded—must be effectively done with the co-operation of the managers of our voluntary hospitals and of the great Poor-Law infirmaries everywhere. As we show in detail on another page, numerous offers of assistance for the provision of hospital accommodation, by the offering of houses, buildings, and equipments of various kinds, are already forthcoming, and will no doubt increase in number as time passes. But this grateful testimony to the generosity of the citizens cannot affect in any way the added increase to the cost of the voluntary hospitals owing to these offers. Never forget that none of them could be organised successfully unless the voluntary hospitals helped them in the supply of the *personnel*. We have, of course, a great naval hospital at Haslar, and certain military hospitals at Netley and in various parts of the country, but from the point of view of the nation as a whole, the voluntary hospital remains the pivot on which, not only the civilian sick must continue to turn, but also on which the Army and Navy Services must rely for recruits. At the same time, the ordinary work of the hospitals may very easily increase, and the sooner the public recognise this the better, or there will be a loss of income as well as of staff at the very time when more demands upon them are probable.

### A WARNING OF THE NEED FOR PUBLIC SUPPORT.

In view of suggestions that have already been made that various charitable organisations shall immediately devote their entire energies and machinery to the emergency of war, it is already time to utter a word of warning. The British public will undoubtedly win through this greatest crisis of the present generation, but they will do so by an exercise of that deliberation and common sense which is their characteristic, and not by the hot-headed confusion of institutions that have been built up by years of devoted attention and still faithfully subserve the purposes for which they were originally founded. Co-operation between existing charitable organisations and the military Services will lessen the hard task of the latter, and bring comfort and relief to thousands of sick and wounded no doubt; but the wholesale diversion of strength and efforts upon which the hospitals are accustomed to rely would speedily have consequences most disastrous to the civilian population, to say nothing of the difficulties in which it would land those centres of healing and medical education when peace reigns once more. It is generally expected at the large general hospitals in London that the demands to be made upon them during the coming autumn and winter will be greater than ever. It cannot be too clearly borne in mind that just when the hospitals are about to be depleted in every department of service, and consequently will have to increase their expenditure in various ways, they are faced with a probable increase of work that, even with their full complement of workers, would severely tax their resources. Therefore only so long as they are backed up by an intelligent public will the Navy and Army be able to rely upon a sufficient staffing of their medical and ambulance departments.

### THE BATHING OF REFRACTORY INFIRMARY PATIENTS.

One of the minor but real difficulties in the administration of Poor-Law institutions is the treatment of the cantankerous individual who refuses to obey regulations obviously necessary for his own good or for the proper management of the institution. Recent correspondence between the Rochdale Guardians and the Local Government Board illustrates this, and is also of interest because of the curious position taken up by the latter Board. The Rochdale Guardians, as required by the Poor-Law Institutions Order, drew up regulations for the bathing of persons admitted to the workhouse and submitted them to the Local Government Board for approval. Amongst the regulations was the following: "Any person refusing to be bathed, without sufficient reason in the opinion of the medical officer, shall be refused admission, but only on the authority of the master." The Local Government Board declined to sanction this regulation on the grounds that it did not appear to them that a destitute person could be properly refused admission to the workhouse owing to his refusal to be bathed, but that he should be dealt with by being punished as a disorderly or refractory inmate in accordance with Articles 32 and 33 of the Poor-Law Institutions Order. After considering this letter the Guardians decided not to omit the regulation and to point out to the Local Government Board that to admit a person who declined to be bathed without sufficient reason was to concede to him the right to remain in the institution in an unclean bodily condition. The final decision has not yet been received, but it would seem difficult for the Local Government Board to justify the position they have taken up. In the interests of the other inmates anyone who refuses without sufficient reason to be bathed on admission ought obviously to be excluded.

### POOR-LAW INSTITUTIONS AND THE WAR.

The example of the Kingston-upon-Hull Guardians in offering their new infirmary to the Government for the use of the wounded, referred to in our last number, has been quickly followed by other Boards.. The Darlington and Wolverhampton Guardians have each offered 100 beds in their infirmaries, the Swaffham Guardians (Norfolk) have offered thirty, and the Long Ashton Guardians (Somerset) have offered the use of their workhouse school, at present empty.

### POOR-LAW INFIRMARIES AND THEIR CONTRACTORS.

Many contractors to Boards of Guardians are applying to be released from supplying goods at the contract prices owing to the advance in price due to the war. In most cases no clause is inserted on the tender forms issued by the Guardians dealing with this matter, and legally the contract can be enforced. At the same time, it would seem that the matter should be dealt with with some amount of leniency. It is generally felt at the moment that there should be no demand to exact higher prices for most provisions, except perhaps sugar and meat. With regard to drugs, the position is somewhat different, in that these have been all manufactured in Germany, and already supplies are diminishing. One suggestion is that these difficulties might be surmounted by contractors invoicing goods at the contract prices, and when the contract has terminated referring the question of an extra allowance to a court of arbitration consisting of business men. By this means, it is thought, public authorities should obtain supplies at reasonable prices without demanding their "pound of flesh." But surely, in view of the unknown time that the war may last and the need for some present working arrangement to cover future as well as immediate fluctuations in price, would not a statesmanlike solution be for the Poor-Law authorities and the contractors to arrange a compromise that should fairly consider the interests of both? Would not the institutions be in a stronger position now than if, holding the contractors to the letter, they find themselves unable to place contracts later on, when the need may be much greater, through their present strictness? What are our readers' views?

### HOSPITALS AND THEIR FOOD CONTRACTS.

The relative position of hospitals and the contractors appears to be settling down on the whole harmoniously. The contractors in many cases have been unable, owing to the first rise in prices, to fulfil the letter of their contracts, and at the end of last week the hospitals' secretaries were facing the problem of what they had better do. No doubt an influencing factor was the date on which existing contracts were due to expire. Some institutions, like the Westminster Hospital for instance, place their contracts for twelve months, from January to December; others, like St. Thomas's, for six months only, and at the last-named the period is from October to April. Mr. Reginald Garratt, Secretary of the Royal Free Hospital, is understood to have favoured keeping his contractors strictly to their legal obligations; but on the other hand, Mr. Walter Alvey, Secretary of Charing Cross, and such hospitals as the Westminster and the Hampstead General, have decided to face the difficulty with more leniency. The argument put forward in favour of this view is that the contracts themselves cannot reasonably be held to have contemplated such an emergency as a European war at the time they were drafted, and that if the hospitals attempted to force their legal claims it is considered doubtful if they would secure a verdict. Supposing they failed to do so, it would mean only bidding against the contractor for supplies, and so the remedy might be worse than the disease. Consequently, the attitude of those more leniently inclined is to say to the contractors, "You do the best you can, and we will treat your bills sympathetically when they come in." The rise in prices is felt, and so far proved, to have been temporary, and the fall, thanks to the Navy and the assistance of the Colonies, will probably go on. Moreover, some hospitals have received very useful gifts in kind since the war was declared, and Westminster Hospital is understood to have two months' supplies of flour, rice, jam, sago, and oats for porridge, from this source.

### THE CROIX ROUGE IN BRUSSELS.

In Brussels all the hospitals conducted by the religious orders and many public buildings are, according to the *Times* correspondent, at the disposal of the Croix Rouge. The arrival both of men and English nurses is reported, and the Duchess of Sutherland is among the volunteers. We may recall in this connection the Hospital of Saint Elizabeth in Brussels, which, on the outskirts, has the reputation of being one of the best equipped in Belgium. It is now full of wounded from the battlefields of last week.

### THE USE OF CHLORINE IN HOSPITAL LAUNDRIES.

Chlorine is employed in the laundries of many asylums and hospitals for the cleansing and sterilisation of soiled clothing. Its advantages are, however, probably not so widely known as should be the case. An apparatus for the production of hypochlorite of sodium by the electrolysis of brine has been in use in the Bexley Asylum laundry for some time and its efficiency has recently been reported upon by Dr. Faulks, the medical superintendent. He says: "The bleaching fluid had a high bactericidal power sufficient practically to sterilise all the clothing treated. All ordinary stains were removed and no injury occurred to cotton or linen fabrics. A considerable amount of time and labour was saved by this method of treating fouled clothing." The method would certainly appear to be worthy of more general adoption.

### A LONDON HOSPITAL PRESIDENT'S EXAMPLE.

We have had this week to hold over a letter from the Grand Duke Michael with reference to the action of the Hampstead General Hospital, of which he is president, in placing beds at the disposal of the soldiers. But the Duke's personal example is worth quoting. He visits his hospital on most days of the week to see that arrangements for a possible emergency are going forward well, and the Grand Duchess, with her two daughters, is attending to gain some personal experience in first-aid work, bed-making, and bandaging. The Grand Duke's private house is being converted. In addition to the forty beds which the hospital has placed at the Government's disposal, the out-patient department, which is no longer in use, is being cleaned out and whitewashed, with a view to some twelve or more beds being placed there also. It is gratifying to note that a generous subscriber, on the announcement of the hospital's offer to the War Office, came forward and presented a cheque to defray all the hospital's outstanding expenses.

### ANTI-TYPHOID INOCULATION AND THE ARMY.

In an official notice issued by the War Office the attention of the officers of the Royal Army Medical Corps is drawn to the importance in the present juncture of securing inoculation, so far as possible, of every member of the Army. However effective may be the sanitary precautions, it is vain to hope that they will be completely successful. No skill or foresight can eliminate the risk that in so large a body of men there may not be some in the incubation stage of the fever when they join the Army. Even more concealed is the danger from unsuspected typhoid carriers. And yet, again, there is the chance of the disease among the inhabitants of the country where the troops may be sent. There is the strongest possible evidence of the preventive value of inoculation. It has been extensively tested in India, and has commanded general confidence. At present only a small percentage of our soldiers enjoy this protection, and as the injection often renders a man unfit for duty for twenty-four to forty-eight hours, it cannot now be provided on anything like a large scale. But special circumstances may be taken advantage of to increase the numbers of the immune. Thus, when a man is temporarily disabled by some minor ailment, he should be inoculated; and even a larger enterprise is possible when it is known that troops will not be actively employed for a time. Here a considerable proportion of them may receive protection. All this goes to show once more how large is the bearing of medicine on the affairs of modern war. Skilled research in the laboratory concerns the efficiency of armies just as it rendered possible the construction of the Panama Canal.

### THE WAR OFFICE AND NURSING HOMES.

If voluntary hospitals and private houses are tumbling over each other with offers of beds for the sick and wounded, it must not be supposed that pay hospitals and nursing homes have stood aside. More than one of the chief of these in London have offered to place at the disposal of the War Office beds for the use of wounded officers, but the truth seems to be that the War Office cannot yet say how many of these it is likely to want. That the supply of beds offered exceeds any reasonably to be foreseen demand appears certain, and probably if a list of offers, excluding those which were obviously marked out for refusal, were made, there would be found enough hospital accommodation for the whole British Army. In addition to these, moreover, as was the case during the South African War, several offers have been made of private hospitals for officers by ladies willing and able to provide the equipment necessary to convert private houses for this purpose.

### THE ABUSE OF THE RED CROSS.

We have grown so much used to seeing the Red Cross badge that, being out of the zone of active hostilities, we forget that all experience shows that it is habitually abused, either from motives of panic, or vague enthusiasm, or even for purposes of treachery. Sir John Furley therefore has publicly pointed out the need for the issue by the War Office of *official* armlets, stamped with a distinguishable mark that can be easily recognised, and that these armlets should be strictly limited to those persons who are actually engaged in hospital or ambulance work ashore or afloat. As it is, he argues, anyone and everyone manufactures Red Cross signs, and uses them with such complete recklessness that he quotes the instance of those who affix Red Crosses to their houses with the intention of showing their willingness to receive wounded—and to protect themselves from hostile acts as well. There is, in short, no doubt that the Red Cross badge can be abused by friends as well as by foes, and that only a few cases of abuse would be sufficient largely to destroy its value as a protection to certain classes of non-combatants.

### POOR-LAW OFFICERS AS VOLUNTEERS.

It is not only the voluntary hospitals from whom offers of service are made and accepted, as the following instance will show. At a meeting of the Islington Board of Guardians held this week a letter from Dr. J. J. A. Sherry, L.R.C.P., L.R.C.S. Edin., district medical officer and public vaccinator, was considered. In view of the great demand for medical men in the Army and Navy, Dr. Sherry desired to know if the Guardians were willing to keep his Union appointments open for him should be volunteer for active service. The Guardians unanimously agreed to accede to Dr. Sherry's request, and the Chairman, on behalf of the Board, wished him "good luck" and safe return. An application was also considered from Mr. Juleff, pharmacist at the Islington Infirmary, on similar lines, and the Board agreed that should this officer volunteer his post should be kept open for him. Again, the Hampstead Board of Guardians have agreed to pay half-salary to dependants of officers on active service and reinstate these officers on their return.

### NEW SECRETARY OF MANCHESTER CHILDREN'S HOSPITAL.

As we go to press we are officially informed that Mr. W. Murray Humphry, chief clerk of the Manchester Royal Infirmary, has been appointed Secretary of the Manchester Children's Hospital, Pendlebury, in succession to Mr. H. J. Eason, who, as we recently announced, has come to London to take up the post of Secretary at the London Lock Hospital.

### FROM PRIVATE HOUSE TO HOSPITAL—A MISTAKEN POLICY.

We have often commented on the growing popularity of the country hospital—like that at Heswall under the care of Mr. Robert Jones, F.R.C.S.—and the passing of the closed ward. The recent offers of hospital quarters from all parts of England, mostly resolving themselves into the suggested conversion of private houses, has prompted Professor Robert Saundby, of Birmingham, to remind the public that wounds, like pulmonary consumption, heal much better in the open air. He therefore urges that the policy of converting lay buildings to hospital purposes is a mistake—as surely the commercial nursing home provides sufficiently glaring an illustration—and contends that our military authorities should remember the well-known experience of Florence Nightingale in this respect, and insist that "suitable sheds should be put up in the parks and pleasure grounds surrounding the buildings it is proposed to appropriate, which latter may well serve the useful purpose of administration blocks." He points out that not only would expense and damage to the offered buildings be minimised, but also that it would clearly be much better for the cases themselves. If the majority of the offers are really patriotic we have no doubt that his reminder will be acted on.

### THE OFFER OF THE FRENCH HOSPITAL.

The committee of the French Hospital, situated in Shaftesbury Avenue, which is open to all French-speaking foreigners, have offered to place at the disposal of the War Office and the Admiralty twenty-five beds at the hospital in London and fifteen at the convalescent home at Brighton, for the accommodation of wounded soldiers or sailors, more especially any who speak French. Possibly a greater number might be made available if there should be need.

### THE USE AND ABUSE OF AMBULANCE SHIPS.

Detailed particulars of the accepted offers of ships for the conveyance of the wounded are coming in now that the Board of Admiralty has ordered local Naval authorities to examine and decide upon all appropriate vessels. It is perhaps worth while to recall that the term "hospital ship" is really a misnomer, implying as it does the regular treatment and accommodation of the wounded. The hospital ship is really only a sea ambulance, and how useful some of the accepted offers are may be seen from the following examples. Lord Tredegar's *Liberty*, the acceptance of which we announced last week and which, we understand, has already been fitted out, is a vessel of over 1,500 tons, 268 feet in length, with a beam of 35 feet 6 inches. Hardly less large is Mr. Loeffler's *Albion*, of 1,300 tons; and another interesting fact is the chartering by Lord Dunraven of a yacht, the *Greta*, for the use of the Australian voluntary hospital. Admiral Beatty's *Sheelah*, Mr. A. M. Tree's *Adventuress*, and Mr. Farquhar's *Medusa* are also in requisition. At the time of the South African War we inspected the hospital ship *Maine*, and learned from it how carefully private offers have to be inspected if the ignorance of hospital conditions is not completely to destroy the good intentions of the donor. That is really the practical argument against such donors being given posts and quarters on these vessels. As laymen and laywomen they do not understand that all the space available must be placed at the disposal of the sick to secure adequate ventilation and air-space. Where the donors are permitted to remain on the vessel too often their comfort is placed before that of those they intend to help, and quarters are given to them amidships which should be reserved rigidly for hospital purposes. We therefore hope that the Naval authorities, who have to examine and report upon the offers made, will make it a condition that no donor, no matter of what age or sex, who has not received prior to the war a medical or a nursing training shall be allowed to accompany their vessel on active work. They can do little good, and the harm they may do is immeasurable.

### CO-OPERATION OF THE WESTERN HOSPITALS.

Supplementing the information that we published last week, we are informed that at the Bristol Royal Infirmary the charming group of buildings constituting the nurses' homes and the whole of the remainder of the establishment on the north side of the infirmary have been given up to the service of the nation. This comprises 426 beds, making a total of 520 beds, and also housing accommodation for about 100 men, rank and file. The dispensary and kitchens on this side of the building are also to be used for the military service, and if and when it becomes necessary the infirmary authorities have made arrangements for the handing over of the old infirmary with another 250 beds. A number of civilian patients have been drafted to Gloucester Infirmary and other neighbouring county hospitals. The number of in-patients (civilians) being treated at the Bristol Royal Infirmary at the present time is 165, and the out-patient department is in full swing.

### CATERPILLARS AND BUBONIC PLAGUE.

The bubonic plague, which has for upwards of twenty years been an annual scourge in Hongkong, has this year proved an exceptionally severe visitation. It is a curious fact that, in spite of all the drastic sanitary measures resorted to by the colonial authorities in cleansing the city of Victoria and the untiring efforts made to rid the houses of rats, the disease recurs year by year with unfailing regularity. It is satisfactory to note, however, that there was in June a steady decline in the number of cases, but the total has been not less than 2,000, a number only exceeded in 1894, when 2,547 persons fell victims to this terribly fatal disease. Commenting on these returns, the *Hongkong Daily Press* draws attention to a rather singular coincidence—viz., the occurrence of a plague of caterpillars in the colony in both of the years named. An extra vote of $1,300 was asked at a recent meeting of the Legislative Council for the Forestry Department, when it was explained that nineteen tons of caterpillars had been collected, and the work of collecting the insects was still not completed. This was the worst visitation, it was added, since 1894, when no less than thirty-six tons of caterpillars were collected. "Here," says the paper above quoted, "we have clear evidence that in the two years when the epidemics of bubonic plague have been most severe in the colony there has been an extraordinary visitation of caterpillars. It would be interesting to know whether this remarkable coincidence has been observed by the authorities, and whether any medical research has been done with a view to ascertain what, if any, relationship exists between these two noteworthy facts." Our contemporary does not say whether the same conditions have prevailed in Canton and other places in Kwangtung, but on the face of it there would seem to be ground for an inquiry by experts.

### THE KERBY PRIVATE WARDS AT NEWBURY.

The latest addition to the pay-ward movement was the recent opening of two private wards at the Newbury District Hospital. The two wards, which form a new wing, have been built as a memorial to Dr. R. J. Kerby, a well-known and very respected practitioner, who has done much useful work both for patients and for the hospital. The opening was performed by Lady Carnarvon, and Mr. Borgnis, the honorary secretary and architect, presented her with a key. The pay-ward movement is steadily progressing, and we hope before long that in fact, if not in name, the American system of classified wards for different economic classes will be established in every district.

### THE DRUG MARKET AND PANIC ORDERING.

Business is proceeding as well as could be expected under the circumstances, and a check has been put upon panic ordering. Advanced prices are necessarily maintained, and in the case of drugs of which stocks cannot at present be replenished still further advances are, of course, highly probable. The only course to pursue is to endeavour to cover immediate requirements. With a view to husbanding the supply of drugs and of preventing over-buying, the Pharmaceutical Society has been in consultation with various Government Departments, and steps are being taken to alleviate the situation as far as possible. In view of the large number of drugs which are affected by the war, it is hardly necessary to single any out for particular mention, more especially as prices are liable to such frequent fluctuations.

### TO OUR READERS.

Contributions are specially invited from any of our readers to these columns. They should deal with topical subjects and news. They must be authenticated for the information of the Editor only. The minimum payment if published is 5s. There is no hard-and-fast rule as to space, but notes of about twenty lines in length are preferred.

